# Haze pollution and urban sprawl: An empirical analysis based on panel simultaneous equation model

**DOI:** 10.1371/journal.pone.0296814

**Published:** 2024-02-29

**Authors:** Luping Huo

**Affiliations:** College of Economics and Finance, Xi’an International Studies University, Xi’an, China; INDIA

## Abstract

Based on the panel data of 227 prefecture-level and above cities in China from 2002 to 2018, a panel linkage equation model is constructed to explore the bidirectional influence relationship between haze pollution and urban sprawl, and the results of the study find that, firstly, there is a bidirectional promotion of causality between haze pollution and urban sprawl. That is, PM2.5 not only has a significant positive effect on urban sprawl, but also urban sprawl has a significant positive correlation with haze pollution, which is further strengthened by adding the air flow coefficient instrumental variable. Second, the heterogeneity analysis yields that haze pollution has different effects on urban sprawl in different regions. Under the sub-regional samples, haze pollution and urban sprawl have a bi-directional significant negative impact relationship in the eastern region, none of the haze pollution and urban sprawl have a bi-directional significant impact relationship in the western region, but both the central region and the northeastern region have a significant positive impact relationship. Under different city sizes, haze pollution and urban sprawl in large, medium and small cities have a bi-directional significant positive impact relationship, and from the numerical size, the degree of influence of haze pollution on urban sprawl in large cities is greater than that in small and medium cities; while the degree of influence of urban sprawl on haze pollution in medium cities is greater than that in large and small cities. Accordingly, it is proposed that urban governance should be adapted to local conditions, focus on innovative technologies to reduce energy consumption, and utilize big data to manage cities.

## 1. Introduction

Since 1978, with China’s booming economic development, the city scale has been expanding, the number of cities has been increasing, the level of urbanization has been rapidly advancing, the spatial structure of the city has changed from monocentric to polycentric, the city has been gradually expanding outward, and the urban sprawl has gradually aroused widespread concern in the academic community. The general viewpoint of the academic community on sprawl is that sprawl is the rapid overexpansion of urban space, the scope of the central area spreads to the periphery, and the urban form is characterized by dispersion, low density, disorder, etc. In fact, this viewpoint is mostly for foreign cities. In fact, this view is mostly a quotation of the concept of urban sprawl for foreign countries, as far as China is concerned, urban sprawl is not a kind of low-density, disordered, and planar expansion, but presents a form of multi-center agglomeration in urban space, and this multi-center agglomeration contributes to the improvement of urban productivity (Wei et al., 2016 [1]). Most of the studies on urban sprawl focus on the impact on environmental pollution and urban productivity (Qin et al., 2016 [2]; Liu, 2020 [3]), or explore the factors affecting urban sprawl, including industrial development, population size, housing price increase, and high-speed rail construction (Wang and Xie, 2016 [4]; Deng and Wang, 2018 [5]; Liang and Wang, 2019 [6]). There are more studies on urban sprawl affecting environmental pollution, and most scholars believe that urban sprawl is an important factor contributing to environmental pollution (Yang and Yan, 2021 [7]). And haze pollution is crucial for human health and social sustainability (Gan et al.,2021 [8]; Ingole et al.,2022 [9]). The 2013 report "Toward an Environmentally Sustainable Future National Environmental Analysis of the People’s Republic of China", jointly published by the Asian Development Bank and Tsinghua University, pointed out that 7 out of 10 of the world’s most polluted cities are in China. In 2016, Yale University released its Environmental Performance Index Report, which again pointed out that China is already the world’s PM2.5 hardest-hit area, with its air quality ranking at the bottom of the list of 180 participating countries [10]. Although the Chinese government has been trying to improve the current situation of haze pollution, its haze pollution is still severe (Chang et al., 2022 [11]).

In fact, there is a certain dual impact mechanism between urban sprawl and environmental pollution. On the one hand, urban sprawl develops over time and exacerbates haze pollution through fixed asset investment and foreign direct investment (Zhang,2021 [12]). On the other hand, haze pollution affects urban sprawl by changing residents’ behavior (Lu et al.,2022 [13]). And from the existing literature, most of the literature has studied the impact of urban sprawl on the environment, while less research has been done on its reverse mechanism. Therefore, in what ways are the mechanisms of haze pollution and urban sprawl interaction realized? This is a question to be studied and explored in depth, and the existing studies do not provide explanations and proofs for this question.

Taking the panel data of 227 prefecture-level and above cities in China from 2002 to 2018 as research samples, this paper utilizes the satellite-monitored PM2.5 concentration to characterize the degree of haze pollution, and constructs the urban sprawl composite index based on the spatial distribution data of the Land Scan population to explore the mechanism of the interplay between haze pollution and urban sprawl. Specifically, the main contributions of this paper are: first, most of the existing literature discusses the influence mechanism of urban spread on haze pollution, while less research on the reverse mechanism of haze pollution on urban spread, this paper is based on this, to explore the bidirectional influence mechanism of haze pollution and urban spread, and clarify the logical relationship between the two. Second, this paper examines the heterogeneous effects of haze pollution and urban spreading in different regions and city sizes based on panel data from 227 prefecture-level and above cities in China. Third, the air mobility coefficient is adopted as an instrumental variable for haze pollution to strengthen the control of endogeneity problem and obtain more robust empirical results.

The remainder of the paper is structured as follows: the second part reviews the relevant literature; the third part analyzes the theoretical mechanisms of haze pollution and urban sprawl, and puts forward the research hypotheses of this paper; the fourth part introduces the data and the model construction; the fifth part is the results of the empirical analysis and the discussion; and the sixth part is the main conclusions and recommendations of this paper.

## 2. Literature review

Although the relationship between haze pollution and urban sprawl has been explored in the literature, the vast majority of the literature focuses on the unidirectional impact of urban sprawl on haze pollution and ignores the mechanism of the impact of haze pollution on urban sprawl. In fact, haze pollution also has a certain impact relationship on urban spreading, especially from the social level, haze pollution brings serious harm to people’s health, leading to a significant increase in the incidence of disease, which in turn promotes the movement of the population to the periphery and the outward expansion of the urban space (Lelieveld et al, 2015 [14]; Fang and Liu, 2016 [15]). Specifically, the relevant studies on urban sprawl by scholars at home and abroad mainly include the following aspects:

### 2.1. Relevant studies on factors affecting urban sprawl

Wang and Xie (2016) [4] concluded that the price increase of housing prices, especially residential commercial housing and commercial business housing, drove urban sprawl. Liu et al. (2018) [16] concluded that the expansion of land size, industrial development and transportation factors are the main reasons for promoting urban sprawl, while the increase in the level of residents’ income does not have a significant impact on urban sprawl. Deng and Wang (2018) [5] found that the development of high-speed railroads is an important cause of urban sprawl, especially for the eastern region of China, large cities and cities with high-speed rail stations located in the periphery of the city have the greatest impact. Liang and Wang (2019) [6] took 104 cities in the Yangtze River Economic Belt as an example and concluded that the development of the secondary industry promotes the intensification of urban sprawl, which is more obvious especially in small and medium-sized cities, while the development of the tertiary industry, especially the living service industry, slows down urban sprawl. Chen (2019) [17] pointed out that urban sprawl has an inverted U-shaped nonlinear impact relationship on the urban-rural income gap. Liao and Wang (2019) [18] pointed out that urban sprawl hinders the high-quality development of urban economy, especially the promotion of the development of secondary industry. Mao and Lu (2019) [19] pointed out that urban sprawl can increase the burden of local public finance, especially the impact on central and western, small and medium-sized cities is more obvious. Li and Wang (2020) [20] point out that urban sprawl inhibits industrial upgrading in the short term, but promotes industrial upgrading in the long term, and the two show a certain non-linear relationship. Zhang et al. (2022) [21] point out that the urban growth target constraint will lead to the local governments placing emphasis on land, ignoring population and investment bias in their emphasis on construction, and ignoring public Tian and Mao (2022) [22] found that regional integration had a significant positive effect on promoting urban sprawl. Tian and Mao (2022) [22] found that regional integration had a significant positive effect on promoting urban sprawl in the YRD region.

### 2.2. Study on haze pollution and urban sprawl

First, about the relationship between the impact of urban sprawl on environmental pollution. Qin et al. (2016) [2] pointed out that urban sprawl will exacerbate haze pollution, especially the spread of small cities will lead to more serious air pollution. Chen et al (2018) [23] concluded that although urban sprawl can exacerbate environmental pollution, the degree of environmental pollution can be reduced through industrial structure upgrading and optimization. Fang et al (2015) [24] found that an increase in "urban continuity" can reduce carbon emissions, but an increase in "urban shape complexity" can reduce carbon emissions. Complexity of urban shape" can promote carbon emissions, and compact urban form can effectively reduce carbon emissions. Fan et al (2018) [25] argued that the increase of urban density and continuity can effectively reduce air pollution, which is due to the reduction of commuting distance and traffic pollution by the compact urban form. Rodriguez et al (2016) [26] showed that the spatial structure of urban space has a significant impact on the spatial distribution of environmental pollution, and areas with high population density are prone to become concentrated areas of sulfur dioxide emissions. The spatial structure of urbanization not only affects urban energy consumption, but also has an impact on urban air quality (Bandeira, 2011 [27]). Liu (2020) [28] pointed out that urban sprawl not only has a direct impact on haze pollution, but also has an indirect impact on haze through transportation patterns and housing styles, among others. Second, about the relationship between the impact of environmental pollution on urban sprawl. Environmental pollution directly affects the health of workers and reduces the quality of human capital and labor productivity (Zivin J, 2012 [29]), as well as reduces the labor supply of workers (Hanna R, 2011 [30]). Air pollution aggravates urban sprawl by inducing discrete industrial distribution and decreasing labor supply, stimulating real estate developers’ investment and land finance. Air pollution also inhibits urban sprawl. Air pollution also inhibits urban sprawl by forcing industrial upgrading and increasing resident health demand (Lu et al., 2022 [13]).

Chinese scholars and foreign scholars study the problem of urban sprawl from different perspectives, and the following deficiencies can be found from the existing research on the relationship between haze pollution and urban sprawl. First, scholars in the past have paid more attention to the impact of urban sprawl on haze pollution, and fewer scholars have studied the impact mechanism of haze pollution on urban sprawl. Second, for the urban sprawl index most scholars use the ratio of the growth rate of the built-up area of the city to the growth rate of the population, and fewer scholars use the analysis of the fine units of population distribution within the city (Fallah et al., 2011 [31]), which is amended by Chinese scholars to construct a new set of research methods by adding the area of the internal area of the city (Qin et al. 2016 [2]), but most of their studies focus on urban sprawl at the provincial level, and the extent of sprawl in Chinese cities at the prefecture level and above is not analyzed. It is based on the above research deficiencies that this paper analyzes the interaction mechanism between haze pollution and urban sprawl using panel data from 227 prefecture-level and above cities in China. It is of great practical significance for the correct understanding of the influence mechanism of urban sprawl, making accurate spatial development planning for Chinese cities, and grasping the future development direction of Chinese cities.

## 3. Theoretical mechanisms and research hypotheses

### 3.1. Impacts of urban sprawl on haze pollution

Urban development is affected by the two effects of urban agglomeration and diffusion. When the agglomeration effect is greater than the diffusion effect, the city is in the stage of agglomeration development, at which time the population gathers to the city center; the industrial layout is more concentrated; the industrial structure is mostly shifted to the secondary and tertiary industries; and the socio-economic development is faster. When the city center develops to a certain stage, due to the limitation of geographic space, it makes the population scale, industrial development and economic development speed are subject to certain limitations. When the city in a saturated state, the diffusion effect is gradually greater than the agglomeration effect, presenting the central area of the city to radiate outward and spread to the surrounding areas. First, at the early stage of spreading, the industrial space layout is chaotic, and the plant construction planning is unreasonable, leading to a decline in the level of rational land use; most of the heavily polluted industrial industries migrate to the periphery of the central city, leading to increased pollution in the spreading area. Secondly, the spread of the population outflow, most of the outsiders live in the suburbs or areas around the central city to reduce the cost of living; industrial relocation has also driven part of the employment population to the suburbs, resulting in increased population density in the suburbs, population management is difficult, the phenomenon of indiscriminate construction and development is serious, but also makes the land use is not centralized, resulting in waste and pollution. Third, with the speed of economic development, per capita income level increases, living in the suburbs or urban areas around the number of private cars owned by the people will also increase, while the suburbs are inconvenient transportation, poorly designed, resulting in traffic congestion, vehicle emissions from the tailpipe caused by environmental pollution. Fourthly, with the out-migration of population, people’s demand for suburban housing increases, and real estate developers build more housing to meet people’s needs, and at the same time, they need to increase the provision of infrastructure and basic public services, which leads to an increase in the demand for high-energy-consuming and high-polluting products, such as cement, coal, iron and steel, and thus exacerbate environmental pollution (Hao, 2014 [32]).

### 3.2. Impacts of haze pollution on urban sprawl

Haze pollution also has certain reverse influence mechanisms on urban sprawl. First, haze pollution can significantly reduce the level of urban development and limit the effective urban agglomeration effect (Au and Henderson, 2006 [33]; Hanlon, 2016 [34]), which in turn leads to outward diffusion of cities and the formation of sprawl. Second, haze pollution is mostly caused by air pollutants emitted by industrial enterprises, thus, the government will choose to force the relocation of these heavily polluting industrial enterprises, or certain industrial enterprises choose to move outward to the suburbs in order to reduce costs, and the layout of enterprise siting spatially results in the outward expansion of the city. Thirdly, the haze contains a large number of harmful substances that jeopardize human health, and people tend to choose the suburbs or rural areas with a better environment in order to enjoy a better living and living environment. Population outflow or increased population density in suburbs will further promote the development of suburbs and the expansion of urban spatial structure. Fourth, haze pollution reduces the level of labor force employment, prompting employed people to choose suburban areas. Most of the employed people are highly mobile agricultural transferees who choose peri-urban areas in order to reduce the cost of commuting and housing, which in turn increases the demand for housing and real estate development and construction, resulting in urban sprawl. In addition, the degree of haze pollution in eastern China is greater than that in central and western China, and haze pollution is characterized by regional heterogeneity (Chang et al., 2022 [11]).

**Fig 1:**
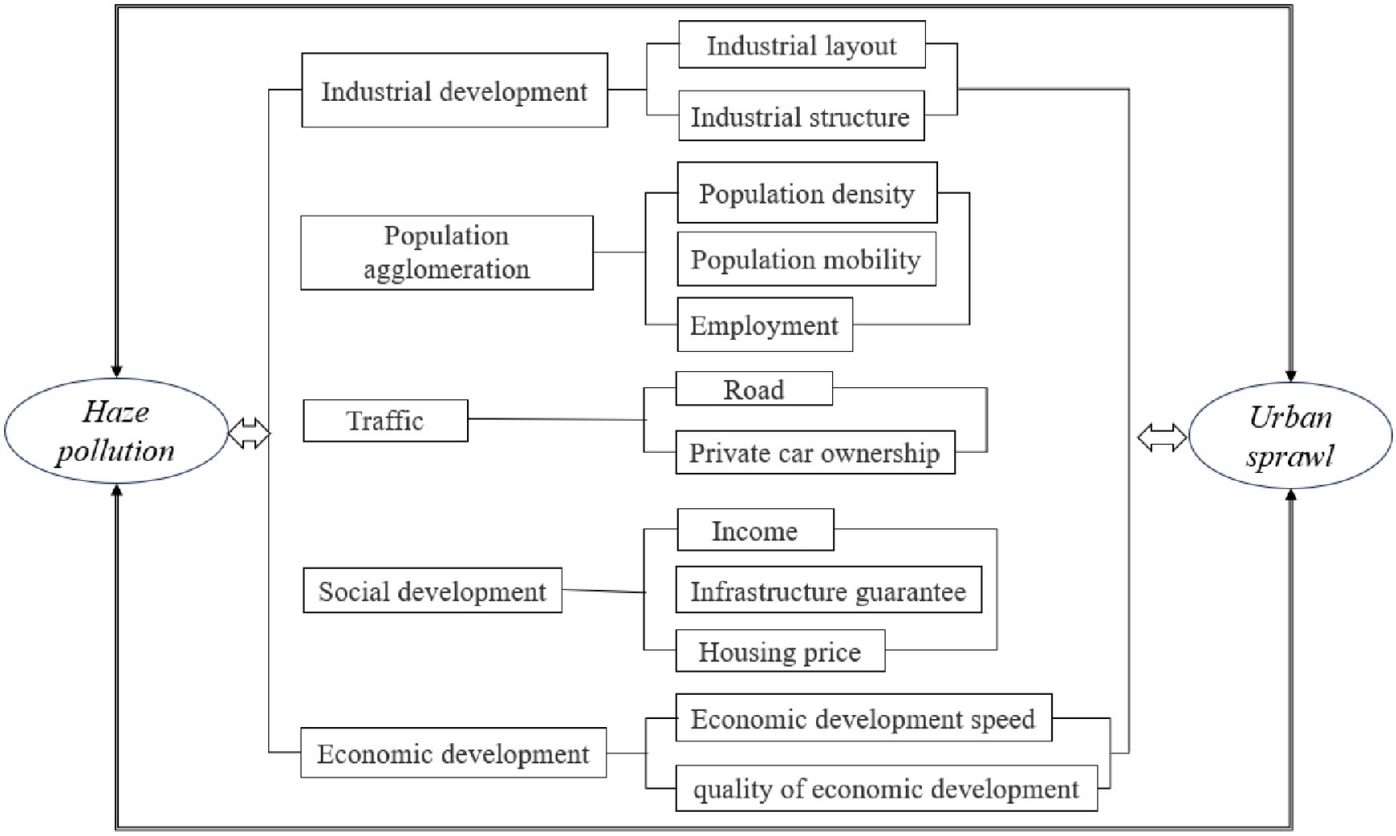
Mechanisms affecting haze pollution and urban sprawl.

Based on the literature review and the mechanism of influence (see Fig 1) we propose the following hypothesis:

Hypothesis 1: There is a bidirectional causal relationship between haze pollution and urban sprawl.
Hypothesis 2: Haze pollution has a heterogeneous effect on urban sprawl in different regions.

## 4. Materials and methods

### 4.1. Variable descriptions and data sources

#### (1) Core variables

*(a) Urban sprawl*. Regarding the urban sprawl index, most scholars use a single indicator to measure it. For example, land and population growth rate (Sierra, 1998 [35]), the difference between the growth rate of built-up area and the growth rate of urban population (Liang and Wang, 2019 [6]), or directly using population density (Fulton et al, 2001 [36]) or residential density (John and Richard, 2003 [37]). Although simple, easy to operate, and with high data availability, single indicators do not allow for a more detailed analysis from the perspective of intra-city space. Fallah et al [31], in 2011, reconstructed the urban sprawl index by dividing the intra-city into a multitude of fine-grained geographic units. As shown in Eq (1).


$$SP_{i}=0.5\times \left(L_{i}-H_{i}\right)+0.5$$ (1)

In Eq (1), *L*_*i*_ represents the proportion of the population whose resident population density is lower than the national average in the small geographical unit within the city. *H*_*i*_ represents the proportion of the population whose resident population density is higher than the national average in the small geographical unit area within the city. The value of *SP*_*i*_ is between 0 and 1, and the larger the value is, the more dispersed the population within the city is, and the more obvious the urban sprawl phenomenon is. Although this index is more accurate than the previous single index, it does not reflect the spatial range differences among inner urban areas. Based on this, Liu (2016) [38] constructed a similar urban-level sprawl index from the perspective of the inner area proportion in 2016, as shown in Eq (2).

$$SA_{i}=0.5\times \left({LP}_{i}-{HP}_{i}\right)+0.5$$ (2)

Where *LP*_*i*_ is the area proportion of the area with population density lower than the national average density in the city, and *HP*_*i*_ is the area proportion of the area with population density higher than the national average density in the city. By integrating the above two indicators, a new spread index is reconstructed, as shown in (3).


$${Sparawl}_{i}=\sqrt{{SA}_{i}*{SP}_{i}}$$ (3)

The urban sprawl index constructed in Eq (3) not only takes into account the density differences of small geographical units within the city, but also compares the degree of differences from the perspective of the inner space of the city, and more accurately depicts the expansion of low-density areas within the city.

Since China does not have detailed statistical data on urban fine geographic units. Therefore, by using remote sensing data, combined with Land Scan global population dynamics monitoring data from 2002 to 2018, we extracted the population distribution data and area data of small geographic units within Chinese cities, and accurately calculated the sprawl index of 227 prefectural-level cities and above in China. The raster resolution of Land Scan’s population distribution data Population of LandScan data download address is: http://landscan.ornl.gov/landscan-datasets is 30", and the data shows the number of permanent residents on each raster, and the total number of populations on the raster is the population size of the city. Land Scan’s raster resolution is 30", while its data shows the number of permanent residents in each raster, and the total number of populations in the raster is the population size of the city.

*(b) Haze pollution*. This paper adopts PM2.5, the most direct indicator reflecting urban environmental pollution, as the explanatory variable, which not only affects urban environmental pollution, but also, causes serious damage to human health. The source of this data is from Atmospheric Composition Analysis Group Download address is: http://fizz.phys.dal.ca/~atmos/martin/? Page id = 140, which is the raster processed data of the mean concentration in mg/m3 after matching the vector map of 227 prefecture-level cities in China.

#### (2) Control variables

In order to verify the effect of haze pollution and urban sprawl, this paper also encompasses some control variables, mainly including:

(a) labor mobility. Directly using the absolute value of the labor force cannot well explain the changes in population mobility, this paper uses the change in the increase in the labor force to better reflect the trend of labor force mobility, so the number of people employed in the labor force is chosen as a proxy variable. It not only reflects the level of growth of the natural labor force in the city, for the population to choose employment after reaching the working age, which is essentially the trend of labor migration (Qin and Zhang, 2019 [39]).
(b) Population density. Population density mainly reflects the degree of population agglomeration in the city, and the development of the city mainly depends on the accumulation of human capital (Shawly, 2022 [40]).
(c) Transportation facilities. The area of urban roads at the end of the year, and the accessibility of urban transportation is the basis of urban development (Zhang et al.2023 [41]).
(d) Opening to the outside world. The amount of actual foreign direct investment, this indicator mainly reflects the development that the introduction of capital elements into the city can bring to the city (Liu and Guo, 2023 [42]).
(e) Indicators of urban economic structure. The proportion of value added of secondary and tertiary industries to GDP. The development of industries can drive the development of urban economy, and then promote urban development (Guo et al., 2023 [43]).
(f) Indicators of residents’ livelihood. Average wage of employed workers. The level of workers’ income implies whether workers are willing to stay in the city or not, and a higher level of workers’ wages implies a greater willingness to stay in the city (Karen, 2018 [44]).

#### (3) Instrumental variables

Air flow coefficient. Considering that haze pollution has a certain spatial diffusion characteristic, we constructed the air flow coefficient value at the prefecture level in China from ERA-INTERIM raster meteorological data published by ECMWF and combined with the atmospheric quantitative model, which was used as an instrumental variable in this paper. The construction method is similar to that mentioned by scholars such as Broner et al. (2012) [45] and Hering and Poncet (2014) [46], and the air flow coefficient construction formula is shown in (4).

$${VC}_{it}={WS}_{it}\times {BLH}_{it}$$ (4)

Where *VC*_*it*_ represents the ventilation coefficients, *WS*_*it*_ represents the wind speed and *BLH*_*it*_ represents the boundary layer height. The original data of wind speed and atmospheric boundary layer height come from the latitude and longitude raster meteorological data released by ECMWF. On this basis, this paper extracts the relevant data of 227 prefecture-level and above cities in China from 2002 to 2018 by using ArcGIS software.

The adoption of air mobility coefficient as an instrumental variable is mainly due to two reasons. First, as the air mobility coefficient increases, the haze pollution level decreases, and there is a certain negative correlation between the two, which satisfies the correlation assumption of instrumental variable selection. The second is that the air flow coefficient is mainly determined by wind speed and the height of the atmospheric boundary layer, both of which are determined by complex meteorological systems and geographic conditions, thus satisfying the exogeneity assumption of the instrumental variable (Broner et al, 2012 [45]). Therefore, this paper draws on the air flow coefficient indicator listed in Chen and Chen (2018) [47] as one of the instrumental variables in this paper.

#### (4) Data source

In this paper, 227 prefecture-level and above cities in China from 2002 to 2018 are selected as our research sample. The reason for choosing 2002 as the beginning of the sample research period is mainly because the urban sprawl index constructed in this paper uses Land Scan global population dynamics monitoring data, which has been monitored mainly since 2002. And the year 2018, the latest available data, is used as the end date of the study. Other data are mainly from the 2003–2019 China Urban Statistical Yearbook and the EPS database. In addition, in order to eliminate the influence of the price factor, data on price-related variables were adjusted to 2002 prices based on the corresponding price index.

Data description and descriptive statistics for the above core variables and all control variables are shown in Table 1.

**Table 1:** Data description and descriptive statistics of variables.

Variable	Index	Sample	Mean	Standard error	Minimum	Maximum
**Core variable**						
Urban Sprawl	Sprawl	3859	0.07	0.18	0	1
Haze Pollution	PM_2.5_	3859	44.12	18.98	4.88	110.12
**Control variable**						
Labor Mobility	Employee	3859	36.76	68.93	1.61	819.3
Urban Agglomeration	Population density	3859	471.83	337	21.2	2702
Traffic facility	Urban road area	3859	1766.06	2363.38	14	21490
Opening Up	FDI	3859	78962.4	188244	12	3082563
Economic Structure	Secondary and tertiary production	3859	87.60	8.58	50.11	100
Residents live	Wage	3859	35557.8	20828.47	5656.28	149843

Note: For some missing data, the geometric mean method was used to calculate the data.

### 4.2. Building models

We constructed a panel covariance model in order to test the bi-directional impact relationship between haze pollution and urban sprawl, so it is shown below:

$$\left\{\begin{array}{lll} Sprawl_{it}= & \alpha_{0}+\alpha_{1}{{PM}_{2.5}}_{it}+\alpha_{2}Density_{it}+\alpha_{3}Road_{it}+\alpha_{4}Wage_{it}+\varepsilon_{it} & \left(5\right)\\ {{PM}_{2.5}}_{it}= & \beta_{0}+\beta_{1}Sprawl_{it}+\beta_{2}Employee_{it}+\beta_{3}ES_{it}+\beta_{4}FDI_{it}+\mu_{it} & \left(6\right) \end{array}\right.$$

In the above formula, *i* represents each city, *t* represents time, *α*_0_ and *β*_0_ are constant terms, *α*_1_-*α*_4_ and *β*_1_-*β*_4_ are coefficients to be estimated, and *ε*_*it*_ and *μ*_*it*_ are error terms. *Sprawl* is the urban sprawl index, *Lmob* refers to labor mobility, and *PM*_2.5_ refers to haze pollution. *Employee* is labor flow, *Density* is population density, *Road* is urban road area at the end of the year, *FDI* is foreign direct utilization investment, *ES* is the proportion of added value of secondary and tertiary industries in GDP, *Wage* is the average wage of working workers.

Eq (5) represents the influence relationship of haze pollution on urban sprawl. Meanwhile, according to the influence mechanism between the two, control variables such as population density, road area and average wage are added.

Eq (6) represents the influence relationship of urban sprawl on haze pollution, and according to the influence mechanism, control variables such as labor flow, the proportion of secondary and tertiary industries and opening to the outside world are also added.

## 5. Results and discussion

### 5.1. Base regression analysis

We processed the panel data according to Eqs (5) and (6) using Stata 15.0 software to obtain the results shown in Table 2. There are two main parts in Table 2: one part analyzes the relationship between the influence of haze pollution on urban sprawl, shown in columns 2 to 4; and one part analyzes the relationship between the influence of urban sprawl on haze pollution, shown in columns 5 to 7.

**Table 2:** Haze pollution and urban sprawl: Estimation results of simultaneous equations.

Variable	*Spawal*			*PM*2.5		
	OLS	Fixed Effect	Random effect	OLS	Fixed Effect	Random effect
*PM*2.5	-0.002*** (0.0002)	0.003*** (0.0003)	0.001** (0.0002)			
*Spawal*				-33.665*** (1.623)	4.665*** (0.986)	3.387*** (0.989)
*Density*	-0.0001***(9.9e-06)	-0.0001*** (0.00003)	-0.0002*** (0.00002)			
*Road*	-3.8e-06*** (1.4e-06)	-0.00001*** (2.3e-06)	-0.00001*** (2e-06)			
*Wage*	1.34e-06*** (1.5e-07)	2.5e-06*** (1.2e-07)	2.3e-06*** (1.2e-07)			
*Employee*				-0.027*** (0.007)	-0.048*** (0.006)	-0.042*** (0.006)
*ES*				-0.034 (0.036)	-0.26*** (0.029)	-0.236*** (0.029)
*FDI*				0.00001*** (2.4e-06)	3e-06** (1.4e-06)	3.1e-06** (1.4e-06)
Cons	0.164*** (0.009)	-0.063*** (0.018)	0.02 (0.015)	49.289*** (3.161)	68.08*** (2.535)	65.902*** (2.717)
R^2^	15.53	11.45	10.81	10.92	0.047	0.047
Year fixed	YES	YES	YES	YES	YES	YES
City fixed	YES	YES	YES	YES	YES	YES
F-test (P)	19.09 (0.000)				89.52 (0.000)	
LM-test (P)			6927.27 (0.000)			18002.59 (0.000)
Hausman-test (P)		95.89 (0.000)			96.36 (0.000)	
Sample	3859	3859	3859	3859	3859	3859

Note:

***, **, * indicate significance at 1%, 5% and 10% significance levels, respectively; The values in parentheses are standard errors.

As a reference, first we conducted a mixed regression on the panel data sample, as shown in column 2 of Table 2. From the results of the mixed regression, it can be seen that haze pollution has a significant negative impact relationship on urban sprawl, i.e., when the PM2.5 emission concentration increases by 1 unit, the urban sprawl decreases by the level of 0.003, when controlling for the other variables to remain unchanged, which is contrary to the basic theory, and the main reason for this result is that we are using panel data, and the different periods of time between each prefecture-level city The main reason for this result is that we are using panel data, and there may be some autocorrelation in the perturbation term between each prefecture-level city in different periods, and the result is caused by the characteristics such as the heterogeneity between each city itself. Since the specifics of each city are not the same and there may be omitted variables that do not vary over time, we consider estimation using a fixed effects model (FE). This is shown in column 3 of Table 2. The fixed effects model results are significantly different from the mixed regression results, and from the final F-test values, we conclude that the FE model outperforms the mixed regression model, that is, it implies that each city has an individual effect. Although the fixed effects indicate that there is some individual effect between each city, this individual effect may still be in the form of a random effect, so we perform a random effect (RE) analysis on the model and obtain the results shown in column 4. As can be seen from the LM test, individual random effects are considered to be present and mixed regressions should not be performed. For the model to determine whether to use fixed or random effects, we conducted a Hausman test, and from the results, we believe that we should use a fixed effects model rather than a random effects model. The FE results show that when controlling for other variables to remain constant, the degree of urban sprawl significantly increases by 0.003 units when the concentration of PM2.5 emissions increases by 1 unit, a finding that suggests that the hazy spreading degree will increase in cities with more severe haze pollution. The other control variables also passed the significance level test, i.e., both population density and road area have a significant negative relationship on urban sprawl, while average wage has a significant positive relationship on urban sprawl, implying that the lower the population density of the city, the less the area of the road construction, and the higher the level of the average wage of the employees, the higher the degree of sprawl, but numerically, the degree of their effects are all relatively small.

Similarly, we further verified the relationship of urban sprawl on haze pollution. As a reference and comparison, we did mixed regression, fixed effect model and random effect model. From the F-test, LM-test and Hausman-test, it can be seen that the choice of fixed effect model is optimal. The results of the fixed effect model show that urban sprawl has a positive correlation on haze pollution, and when the degree of urban sprawl increases by 1 unit, haze pollution will increase by 4.665 units. From other control variables, all the control variables passed the t significance level test, and it can be obtained that when labor mobility is faster and secondary and tertiary industries are more concentrated, the degree of haze pollution is smaller. Compared with labor mobility, the greater the impact of the proportion of secondary and tertiary industries on haze pollution, which is an important source of pollution. From this, it can be inferred that urban sprawl is the result of a combination of factors, and in the process of urban sprawl, the reverse will cause haze pollution.

In summary, this paper verifies hypothesis Hypothesis1, that is, there is a bidirectional causal relationship between haze pollution and urban sprawl, in which haze pollution has a positive influence relationship on urban sprawl. Urban sprawl has a significant positive relationship to haze pollution, and the two promote and influence each other.

### 5.2. Robustness test

In order to test the reliability of the above results, we choose to use the instrumental variable method to test the results of the covariate estimation. After adding the instrumental variable of air flow coefficient in the model, we found that whether it is the relationship of linear influence of haze pollution on urban sprawl or urban sprawl on haze pollution, the resultant coefficients passed the test of significance level, and at the same time the sign is consistent in Table 3. Haze pollution and urban sprawl have significant positive promoting effect on each other. In summary, it can be concluded that the results of the joint equation estimation in Table 2 are robust.

**Table 3:** Results of robustness test.

Variable	*Spawal*	*PM*2.5
*PM*2.5	0.003*** (0.0003)	
*Spawal*		4.763*** (0.986)
Control variables	YES	YES
Cons	-0.148** (0.069)	76.758*** (4.849)
R^2^	11.49	0.048
Year fixed	YES	YES
City fixed	YES	YES
Sample	3859	3859

Note:

***, **, * indicate significance at 1%, 5% and 10% significance levels, respectively; The values in parentheses are standard errors.

### 5.3. Heterogeneity analysis

#### (1) East, Central, West and Northeast regions

The level of economic development in different regions is often inconsistent, and even if the same policies are implemented between regions, the final result is heterogeneous. Therefore, in order to further consider whether haze pollution and urban sprawl are characterized by regional heterogeneity, we will further analyze the relationship between haze pollution and urban sprawl in the eastern, central, western and northeastern regions of China. The eastern region contains 92 cities with a sample size of 1,564. There were 61 cities in the central region with a sample size of 1,037. The western region included 41 cities with a sample size of 697. There are 33 cities in Northeast China with a sample size of 561. The estimated results of the relationship between haze pollution and urban sprawl for the eastern, central, western and northeastern regions of China are shown in Table 4.

**Table 4:** Estimation results of China’s subregional sample.

Variable	East region		Central region		West region		Northeast region	
	*Spawal*	*PM*2.5	*Spawal*	*PM*2.5	*Spawal*	*PM*2.5	*Spawal*	*PM*2.5
*PM*2.5	-0.001*** (0.0004)		0.003*** (0.0005)		-0.00004 (0.0002)		0.011*** (0.003)	
*Spawal*		-6.935*** (1.767)		11.37** (2.213)		3.743 (9.989)		2.074*** (0.671)
Control variables	YES	YES	YES	YES	YES	YES	YES	YES
Cons	0.106*** (0.021)	54.746*** (3.941)	-0.121*** (0.038)	61.666*** (5.221)	0.045*** (0.018)	92.3*** (7.684)	-0.115 (1.78)	17.116*** (2.721)
R^2^	12.83	56.65	24.28	71.48	14.22	57.8	51.42	70.27
Year fixed	YES	YES	YES	YES	YES	YES	YES	YES
City fixed	YES	YES	YES	YES	YES	YES	YES	YES
Sample	1564	1564	1037	1037	697	697	561	561

We constructed mixed OLS regression, fixed-effects model and random-effects model for different regions. Through the test analysis, it is concluded that the fixed-effect model is better among different regions, and thus the estimated results in the table are all fixed-effect model. From the data in the Table 4, it can be found that there are differences in the relationship between haze pollution on urban sprawl in different regions. In the eastern region, haze pollution has a significant negative impact on urban sprawl in both directions, in the western region, haze pollution has no significant impact on urban sprawl in both directions, but in the central region and the northeastern region, there is a significant positive impact on urban sprawl. The possible reason for this result is that the level of urban development in the eastern region is relatively fast compared with other regions. And both the urban development stage and haze pollution management have reached a mature stage, so there is a mutual check and balances between haze pollution and urban sprawl. Compared with other regions, the western region, whether it is the level of economic development or haze management capabilities are relatively backward, by resources, capital, technology, talents and other constraints lead to slower urban development, urban sprawl will inevitably decline, the city is still in the stage of rapid development, most of the western region is a resource-based city, mainly relying on coal and other high consumption, high pollution of resources caused by haze pollution, haze pollution and urban sprawl have not been found to have a mutual influence on each other. The level of urban development in the central and northeastern regions is exactly between the eastern and western regions. The central region is a pie-shaped development, haze pollution will be affected by the spatial spillover effect of the pollution in the surrounding eastern region, resulting in an increase in the concentration of PM2.5. With the rapid development of the industrial enterprises in the central region, the gradual formation of the urban sprawl. For the Northeast, which is mainly an old industrial base, in the stage of rapid industrial development, which will inevitably cause haze pollution. By the geographic location, climate and other impacts, the Northeast region of the outflow of talent; and by the national policy "revitalization of the old industrial base in Northeast ", the impact of the development of new industrial bases, such as the expansion of the land, the spread of the city intensified.

#### (2) Large cities, medium cities and small cities

We follow the different city population sizes, the city population of 1 million or more is considered as large cities, and there are 1,377 large cities in this paper, the city population between 500,000 and 1 million is considered as medium-sized cities, and there are 1,921 of them, and the city population of less than 500,000 is considered as small cities, and there are 561 of them. We further explore the impact relationship between haze pollution and urban sprawl under different city sizes. Finally, the fixed effect model was selected for parameter estimation to obtain the results shown in Table 5. From the table, it can be seen that there is a significant positive impact relationship between haze pollution and urban sprawl in both directions, regardless of whether it is a large city, a medium-sized city or a small city. However, in terms of numerical magnitude, the degree of influence of haze pollution on urban sprawl is greater in large cities than in small and medium-sized cities (0.003>0.002). The degree of influence of urban sprawl on haze pollution in medium-sized cities is greater than that in lager and small cities (5.606>4.753>3.536). The possible reasons for this are the rapid development of large cities, large population size, faster development of industrial industries, which triggers an increase in the degree of haze pollution, and further urban sprawl of the phenomena of population outflow, enterprise relocation, industrial relocation, and rapid land expansion. However, the degree of urban agglomeration of small and medium-sized cities is lower than that of large cities, first of all, the need to realize the rapid development of the economy within the city. Although caused by the increase in the degree of pollution, but will not lead to urban sprawl. However, with the further development of industrial industry and service industry, the rapid rise of resource-based cities will inevitably lead to the outward expansion of the city, presenting the characteristics of the trend of urban sprawl.

**Table 5:** Sample estimation results of different city sizes.

Variable	Large city		Medium city		Small city	
	*Spawal*	*PM*2.5	*Spawal*	*PM*2.5	*Spawal*	*PM*2.5
*PM*2.5	0.003*** (0.001)		0.002*** (0.0004)		0.002 (0.001)	
*Spawal*		4.753*** (1.195)		5.606*** (1.258)		3.536** (1.76)
Control variables	YES	YES	YES	YES	YES	YES
Cons	-0.007 (0.043)	51.543*** (5.728)	-0.020 (0.027)	61.589*** (3.936)	0.188*** (0.067)	34.985*** (3.929)
R^2^	18.79	52.15	16.1	50.42	20.89	53.31
Year fixed	YES	YES	YES	YES	YES	YES
City fixed	YES	YES	YES	YES	YES	YES
Sample	1377	1377	1921	1921	561	561

In summary, we analyze the relationship between the influence of haze pollution and urban spread from the perspectives of four major regions and three different city scales in China. It can be found that the increase of haze pollution is affected by factors such as climate, capital, technology, talents and policies, and has different impacts on the sprawl of different regions and city scales, thus further verifying Hypothesis 2. Which is that haze pollution has a heterogeneous effect on urban sprawl in different regions and scales.

## 6. Conclusions and suggestions

This paper explores the bidirectional causal relationship between haze pollution and urban sprawl and its influence mechanism by constructing a linkage equation model using panel data of 227 prefecture-level and above cities in China from 2002 to 2018, and further explores the heterogeneous influence relationship of haze pollution on urban sprawl under different city sizes in east, central, west and northeast regions of China., large, medium and small cities, and draws the following conclusions: (1) Under the full sample data, haze pollution has a significant positive influence relationship on urban sprawl, and urban sprawl also has a significant positive correlation on haze pollution. Meanwhile, the degree of influence of urban sprawl on haze pollution (4.665) is greater than the degree of influence of haze pollution on urban sprawl (0.003). This conclusion is further strengthened by including the air mobility coefficient instrumental variable. It shows that there is a bi-directional promoting causal influence between haze pollution and urban sprawl. Hypothesis 1 is established. (2) Heterogeneity analysis yields that haze pollution has different effects on urban sprawl in different regions. Under the sub-regional samples, haze pollution and urban sprawl in eastern China have a bidirectional significant negative impact relationship in both directions, while haze pollution and urban sprawl in western region do not have a significant negative relationship in both directions, but the central region and the northeastern region have a significant positive relationship. Under different city sizes, haze pollution and urban sprawl have a bi-directional significant positive relationship, regardless of whether it is a large city, a medium-sized city or a small city. However, in terms of numerical magnitude, the degree of influence of haze pollution on urban sprawl is greater in large cities than in small and medium-sized cities (0.003>0.002). While the degree of influence of urban sprawl on haze pollution is greater in medium-sized cities than in large and small cities (5.606>4.753>3.536). It indicates that haze pollution has a heterogeneous effect on urban sprawl. Hypothesis 2 is established. (3) From the results of the control variables, it can be found that population density and road traffic area will significantly inhibit the degree of urban sprawl. And industrial structure optimization and labor mobility will reduce the degree of haze pollution.

From the above research results, we can see that we cannot ignore that urban sprawl will trigger haze pollution, and at the same time, we should pay more attention to the impact of haze on the urban spatial development pattern. Based on this, the following suggestions are put forward: Firstly, each local governments should adapt to local conditions, judge the stage of urban development according to the level of urban development in the region, and reasonably plan the urban spatial pattern, so as to prevent the phenomena of disorderly expansion of the spreading and indiscriminate development and construction. Secondly, targeting to formulate the system of pollution control can be based on the analysis of the pollution problems that have already appeared in the large cities or the eastern developed regions, and targeting the western regions or small and medium-sized cities, to eliminate the emergence of the problems that have already appeared in large cities, and further realize the sustainable should focus on innovative technology, the use of artificial intelligence, big data and other network information to reduce the waste of finite resources, and the rational use of resource reuse technology to achieve high utilization of resources and low consumption. Finally, modernization of urban governance, focusing on the management mode, the use of accurate data to rationally plan the spatial layout of the city.

## Supporting information

S1 Dataset Data on PM2.5 concentration, urban sprawl and all control variables for 227 cities from 2002 to 2018, corresponding to Table 1. (XLSX)
